# Comparative plastomes of *Pueraria montana* var. *lobata* (Leguminosae: Phaseoleae) and closely related taxa: insights into phylogenomic implications and evolutionary divergence

**DOI:** 10.1186/s12864-023-09356-8

**Published:** 2023-06-02

**Authors:** Yun Zhou, Xiao-Hong Shang, Liang Xiao, Zheng-Dan Wu, Sheng Cao, Hua-Bing Yan

**Affiliations:** 1 College of Pharmacy, Guangxi Medical University, Nanning, 530021 Guangxi China; 2 Cash Crops Research Institute, Guangxi Academy of Agricultural Sciences, Nanning, 530007 Guangxi China

**Keywords:** Kudzu, Plastomes, Phylogeny, *Pueraria*, Genome comparison

## Abstract

**Background:**

*Pueraria montana* var. *lobata* (kudzu) is an important food and medicinal crop in Asia. However, the phylogenetic relationships between *Pueraria montana* var. *lobata* and the other two varieties (*P. montana* var. *thomsonii* and *P. montana* var. *montana*) remain debated. Although there is increasing evidence showing that *P. montana* var. *lobata* adapts to various environments and is an invasive species in America, few studies have systematically investigated the role of the phylogenetic relationships and evolutionary patterns of plastomes between *P. montana* var. *lobata* and its closely related taxa.

**Results:**

26 newly sequenced chloroplast genomes of *Pueraria* accessions resulted in assembled plastomes with sizes ranging from 153,360 bp to 153,551 bp. Each chloroplast genome contained 130 genes, including eight rRNA genes, 37 tRNA genes, and 85 protein-coding genes. For 24 newly sequenced accessions of these three varieties of *P. montana*, we detected three genes and ten noncoding regions with higher nucleotide diversity (π). After incorporated publically available chloroplast genomes of *Pueraria* and other legumes, 47 chloroplast genomes were used to construct phylogenetic trees, including seven *P. montana* var. *lobata*, 14 *P. montana* var. *thomsonii* and six *P. montana* var. *montana*. Phylogenetic analysis revealed that *P. montana* var. *lobata* and *P. montana* var. *thomsonii* formed a clade, while all sampled *P. montana* var. *montana* formed another cluster based on cp genomes, LSC, SSC and protein-coding genes. Twenty-six amino acid residues were identified under positive selection with the site model. We also detected six genes (*accD, ndhB, ndhC, rpl2, rpoC2*, and *rps2*) that account for among-site variation in selective constraint under the clade model between accessions of the *Pueraria montana* var. *lobata* clade and the *Pueraria montana* var. *montana* clade.

**Conclusion:**

Our data provide novel comparative plastid genomic insights into conservative gene content and structure of cp genomes pertaining to *P. montana* var. *lobata* and the other two varieties, and reveal an important phylogenetic clue and plastid divergence among related taxa of *P. montana* come from loci that own moderate variation and underwent modest selection.

**Supplementary Information:**

The online version contains supplementary material available at 10.1186/s12864-023-09356-8.

## Background

*Pueraria montana var. lobata* (Willd.) Sanjappa & Pradeep (2n = 2x = 22) is a semi-woody, perennial liana, which belongs to the Leguminosae family. It is native to Southeast Asia and introduced in Africa, America, and Europe [1]. As an economic crop, it contains puerarin and other functional components and is used in the production of both pharmaceuticals and health foods [2]. Its dried rhizome is listed in the Chinese Pharmacopeia as kudzu [3]. Kudzu was introduced from Asia to America about 150 years ago for soil erosion control and cattle feed, but later became a highly invasive species [4].

**Fig. 1:**
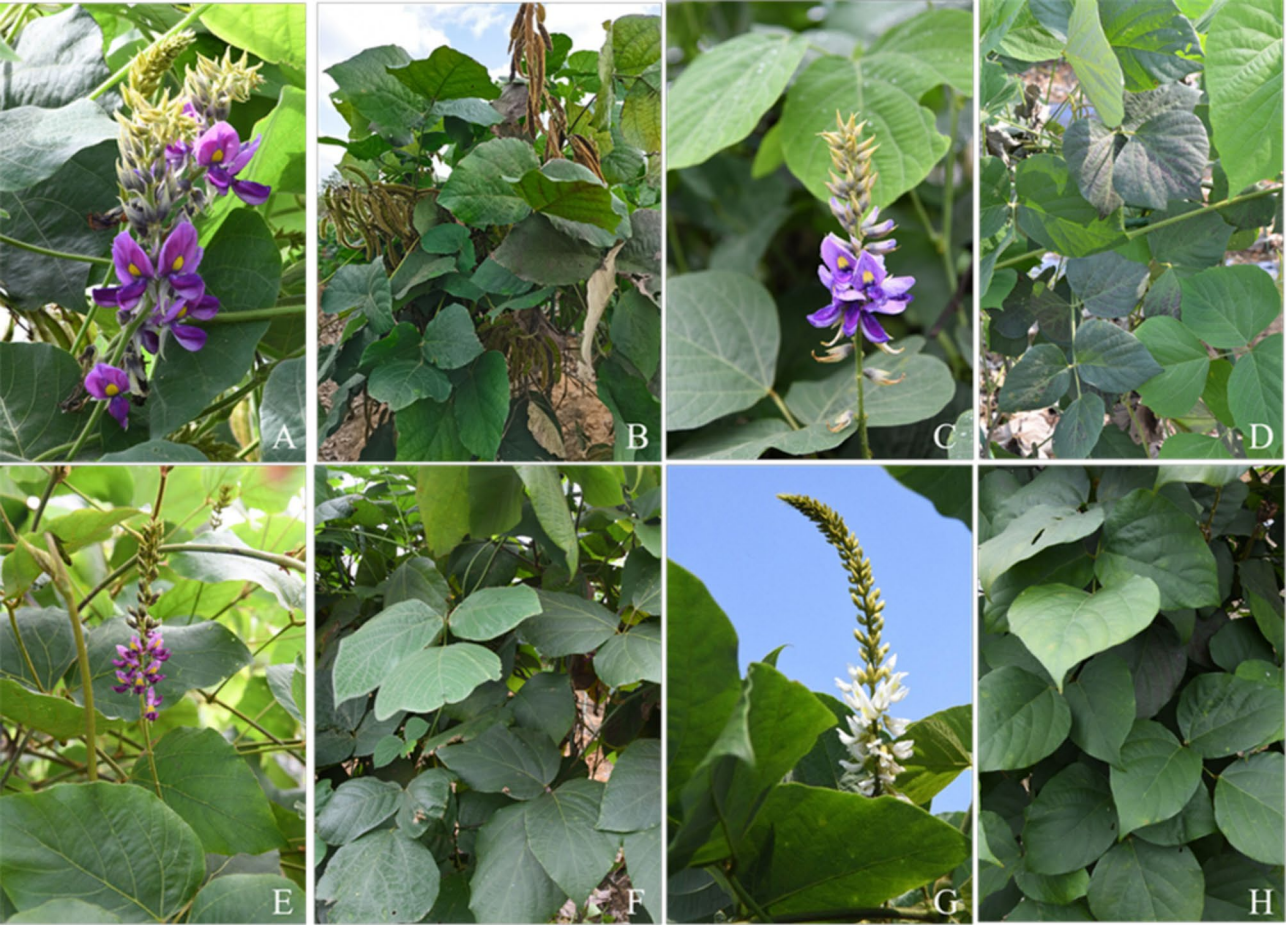
Morphological diversity of *Pueraria montana* var. *lobata* and the other two varieties in this study. **(A)-(B)***Pueraria montana* var. *lobata* (YG_127); **(C)-(D)***Pueraria montana* var. *thomsonii* (FG_43); **(E)-(H)***Pueraria montana* var. *montana* (GMM_96 and GMM_94). The code of the individual is consistent with Table 1

Due to considerable morphological diversity, complex domestication history, and similar health and cosmetic benefits, *Pueraria montana* var. *lobata* and the other two varieties faced a protracted period of taxonomic uncertainty. The dried rhizome of *Pueraria montana* var. *thomsonii* (Benth.) Wiersema ex D.B. Ward (common name “Fenge” in Chinese) is usually used as a vegetable and for extracting starch, while *Pueraria montana* var. *montana* (Willdenow) Maesen & S. M. Almeida ex Sanjappa & Predeep (common name “Gemamu” in Chinese) is confusedly used medicinally and sometimes as a cover crop and fodder [5]. Some taxonomic questions on *P. montana* var. *lobata* and the other two varieties are up for debate because of the phenomena of homonym, unscientific classification, genetic relation confusion, and transitional states of highly variable traits. The synonyms or some combinations of *P. montana* var. *lobata* in the different studies complicated the species delimitation on account of variation in leaf shape, inflorescence and flower size, and geography by different authors (Table S1), which may have further hindered the classification of *P. montana* var. *lobata* and the other two varieties. Several researchers consider that these taxa of *P. montana* were one species with three varieties [5–8], while Ohashi et al. [9] considered that they should be treated as two species. *Pueraria montana* var. *lobata* and the other two varieties share the following diagnostic characteristics [5]: robust climbers with tuberous roots, dorsifixed stipules entire to fringed at the base, trifoliolate, and flowers clustered at each node of the rachis (Fig. 1, Fig. S1, Table S2). The previous study focusing on the samples located in Taiwan showed that *P. lobata* (= *P. montana* var. *lobata*) and *P*. *montana* (= *P. montana* var. *montana*) are single species, while *P. lobata* subsp. *thomsonii* (Benth.) Ohashi and Tateishi is considered the subspecies of *P. lobata* (= *P. montana* var. *lobata*) based on the leaflets, the relative length between inflorescence and leaves, and the relative length between the wing and keel-petal [9]. The morphology of leaflets from entire to trilobed had no quantitative standard among these three varieties we observed in the field (Fig. S1), as well as the width and the length. Furthermore, the range of the length of the pedicel, bracteole, flower, and calyx overlapped among these taxa [6, 9]. For instance, the length of the pedicel of *P. lobata* subsp. *lobata* (= *P. montana* var. *lobata*), *P. lobata* subsp. *thomsonii* (= *P. montana* var. *thomsonii*) and *P. montana* (= *P. montana* var. *montana*) are 2–6 mm, 3–7 mm, and 1.5-4 mm, respectively [9]. Especially, the intermediate morphology of quantitative traits (i.e. the width and length of apex leaflets, the length of pedicel) between *P. montana* var. *thomsonii* and *P. montana* var. *lobata* reveal that it may be questionable to classification and rank of *P. montana* merely based on the morphological characteristics (Table S2) [7, 9]. For example, *P. montana* var. *lobata* and *P. montana* var. *thomsonii* shared the pedicel with white short spreading hairy [9].

Molecular phylogenies are often incongruent with morphologically-based classification schemes, leading to difficulty in understanding the evolution of morphological traits in many crops involving domestication. Several molecular markers have been used to reconstruct a phylogenetic frame of *Pueraria* or legume species, but the phylogenetic relationships between *P. montana* and closely related species are still disputed due to morphological intermediacy and varying molecular markers [7, 10–20]. In previously published works, *Pueraria montana* var. *lobata* and the other two varieties formed a clade and nested within *Pueraria sensu stricto* based on two cpDNA markers, while they formed a polytomy based on one nuclear marker (*AS2*) [16]. *Pueraria montana* var. *montana* should be considered as a single species, and *P. montana* var. *thomsonii* sister to *P. montana* var. *lobata* based on nrITS sequences involving four accessions of *P. montana* and its varieties [11]. *Pueraria montana* var. *thomsonii*, *P. montana* var. *lobata*, and *P. montana* var. *montana* clustered within a monophyletic clade but relationships among these taxa are less resolved based on nrITS sequences of 11 accessions of *Pueraria* [15]. Therefore, insufficiently informative characters and sparse representative accessions in sampling had been the problems for investigating the phylogeny between *P. montana* var. *lobata* and closely related taxa.

Full plastome sequencing is useful in resolving complex evolutionary relationships in closely related species due to more informative sites and lower nucleotide substitution rates [21–25]. Moreover, accumulating studies showed that positive selection may be critical for the evolution of genes in plastomes, even if the structure and sequence of plastomes among closely related taxa are conservative [26–33]. *Pueraria montana* var. *lobata* is distributed widely and become an invasive plant due to the breeding system, favorable climatic conditions, and human-mediated movement [34, 35]. Given the many important biological processes in plastids, including photosynthesis, the assimilation of sulfur and nitrogen, and phytohormones, it is to be expected that the plastome would be diversified or adapted to a specific environment due to natural selection [36].

Here, we sequenced twenty-six chloroplast genomes for *P. montana* var. *lobata* and its allies to explore plastid diversity in the genetic background of kudzu vine that originated from different geographical regions. We aim to (1) establish a phylogenetic framework for *P. montana* var. *lobata* and closely related taxa; and (2) compare the structural variation and positive selection of the chloroplast genomes of *P. montana* var. *lobata* and closely related taxa.

## Results

### General features of Plastomes

**Fig. 2:**
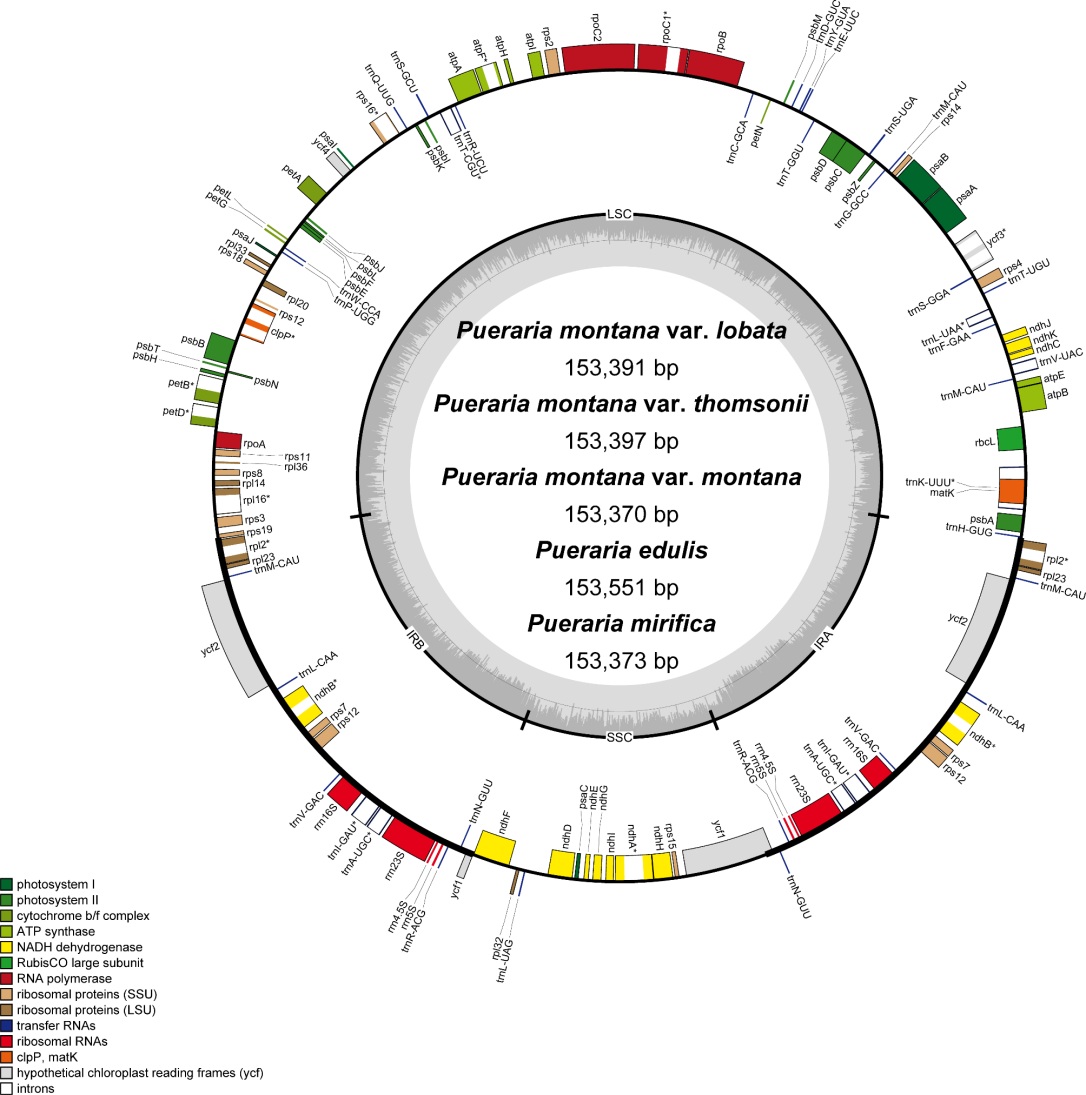
Gene maps of chloroplast genomes of *Pueraria* accessions in the present study. Genes outside the circles are transcribed in counter clockwise direction and those inside in counterclockwise direction. The colored bars indicate known protein-coding genes, transfer RNA genes, and ribosomal RNA genes. The dashed, dark grey area in the inner circle denotes GC content, and the light grey area indicates genome AT content. LSC indicates large single copy; SSC, indicates small single copy and IR, indicates inverted repeat

We sequenced and assembled twenty-six plastomes from *P. montana* and its allies (Table 1, Table S3). All newly sequenced genomes showed typical quadripartite structure, ranging from 153,360 bp (*P. montana* var. *montana*_GMM_112) to 153,551 bp (*P. edulis_*SYG_78) (Fig. 2), which was similar to previously published complete chloroplast genomes of *P. montana* var. *lobata* and *P. montana* var. *thomsonii* [18–20]. The GC content of these plastomes was 35.4%, except for *P. mirifica* A. Shaw and Suvat that was 35.5%. We identified 112 genes comprising four ribosomal RNAs, 30 transfer RNAs, and 78 protein-coding genes. Seven protein-coding genes, four rRNAs, and seven tRNAs are duplicated in the inverted repeat regions, and thus each chloroplast genome harbored 130 genes in total (Table S4). The size of the inverted repeat (IR), large single copy (LSC) and small single copy (SSC) regions ranged from 25,632 bp (*P. montana* var. *montana*_GMM_112) to 25,684 bp (*P. mirifica_*TG_79), 84,077 bp (*P. mirifica_*TG_79) to 84,248 bp (*P. edulis_*SYG_78), and 17,971 bp (*P. mirifica_*TG_79) to 18,005 bp (*P. montana* var. *montana*_GMM_112), respectively.

**Table 1:** General features of plastomes newly sequenced in the present study

Taxon	Voucher Code	Collected sites	Size (bp)				GenBank
			Complete sequences	LSC	SSC	IR	
*P. montana* var. *lobata*	YG_19	Luojiao, Luoping, Guangxi	153,391	84,124	17,987	25,640	OM156439
*P. montana* var. *lobata*	YG_110	Huaihua, Hunan	153,392	84,125	17,987	25,640	OM156447
*P. montana* var. *lobata*	YG_123	Funing County, Wenshan, Yunan	153,392	84,125	17,987	25,640	OM156448
*P. montana* var. *lobata*	YG_124	Dianjiang County, Chongqing	153,392	84,125	17,987	25,640	OM156451
*P. montana* var. *lobata*	YG_126	Beipei, Chongqing	153,392	84,125	17,987	25,640	OM156455
*P. montana* var. *lobata*	YG_127	Longyan, Fujian	153,407	84,139	17,986	25,641	OM135501
*P. montana* var. *montana*	GMM_12	Laibing, Guangxi	153,370	84,114	17,988	25,634	OM135499
*P. montana* var. *montana*	GMM_35	Teng County, Wuzhou, Guangxi	153,415	84,154	17,991	25,635	OM135502
*P. montana* var. *montana*	GMM_94	Xuanwu, Laibing, Guangxi	153,419	84,148	17,991	25,640	OM156459
*P. montana* var. *montana*	GMM_96	Xishuangbanna, Yunnan	153,368	84,119	17,981	25,634	OM156458
*P. montana* var. *montana*	GMM_112	Huaihua, Hunan	153,360	84,082	18,005	25,632	OM156457
*P. montana* var. *thomsonii*	FG_4	Guxiu, Teng County, Wuzhou, Guangxi	153,392	84,125	17,987	25,640	OM156443
*P. montana* var. *thomsonii*	FG_7	Guangxi	153,392	84,125	17,987	25,640	OM156444
*P. montana* var. *thomsonii*	FG_11	Longmenjiang, Nanning, Guangxi	153,391	84,124	17,987	25,640	OM156438
*P. montana* var. *thomsonii*	FG_23	Longzhou, Guangxi	153,397	84,130	17,987	25,640	OM156440
*P. montana* var. *thomsonii*	FG_27	Sizhou, Dexing, Jiangxi	153,392	84,125	17,987	25,640	OM156446
*P. montana* var. *thomsonii*	FG_43	Teng County, Wuzhou, Guangxi	153,397	84,130	17,987	25,640	OM156441
*P. montana* var. *thomsonii*	FG_96	Zhaoqing, Guangdong	153,392	84,125	17,987	25,640	OM156453
*P. montana* var. *thomsonii*	FG_97	Hechuan, Chongqing	153,397	84,130	17,987	25,640	OM156442
*P. montana* var. *thomsonii*	FG_98	Huoshan, Shaoguan, Guangdong	153,392	84,125	17,987	25,640	OM156454
*P. montana* var. *thomsonii*	FG_100	Zhongxiang, Hubei	153,392	84,125	17,987	25,640	OM156452
*P. montana* var. *thomsonii*	FG_101	Pingnan, Guigang, Guangxi	153,392	84,125	17,987	25,640	OM156450
*P. montana* var. *thomsonii*	FG_102	Wuxuan, Guangxi	153,392	84,125	17,987	25,640	OM156445
*P. montana* var. *thomsonii*	FG_103	Pingle, Guilin, Guangxi	153,392	84,125	17,987	25,640	OM156449
*Pueraria mirifica* Airy Shaw & Suvat.	TG_79	Luojiao, Luoping, Guangxi	153,373	84,084	17,965	25,662	OM135503
*Pueraria edulis* Pampan.	SYG_78	Longyan, Fujian	153,551	84,255	17,992	25,642	OM048895

The mVISTA analysis revealed that the chloroplast genomes of *P. montana* var. *lobata* and closely related taxa were conserved generally with a few variable regions (Fig. S2). The proportion of variable sites was higher in the LSC regions than the SSC and IR regions, and higher in the intergenic spacers than the coding regions. We also found there was low variability of adjacent genes in the IRb/SSC and SSC/IRa boundaries of *P. montana* var. *lobata* and the other two varieties (Fig. S3). We did not detect any inversions and large indels either within the varieties of *P. montana* or in comparison to the *P. edulis* and *P. mirifica* (Fig. S4). The range of nucleotide diversity was 0-0.00191, with an average of 0.07839. Three genes had a higher π: *ndhJ*, *ycf1* and *rps15*, and the corresponding noncoding regions *trnK-rbcL*, *trnG-psbZ*, *psbD-trnT*, *trnD-psbM*, *petN-trnC*, *psbK-trnQ*, *rps16-accD*, *psbB-psbT*, *rps3-rps19*, and *ccsA-ndhA* (Fig. S5).

### Plastome phylogenomics

**Fig. 3:**
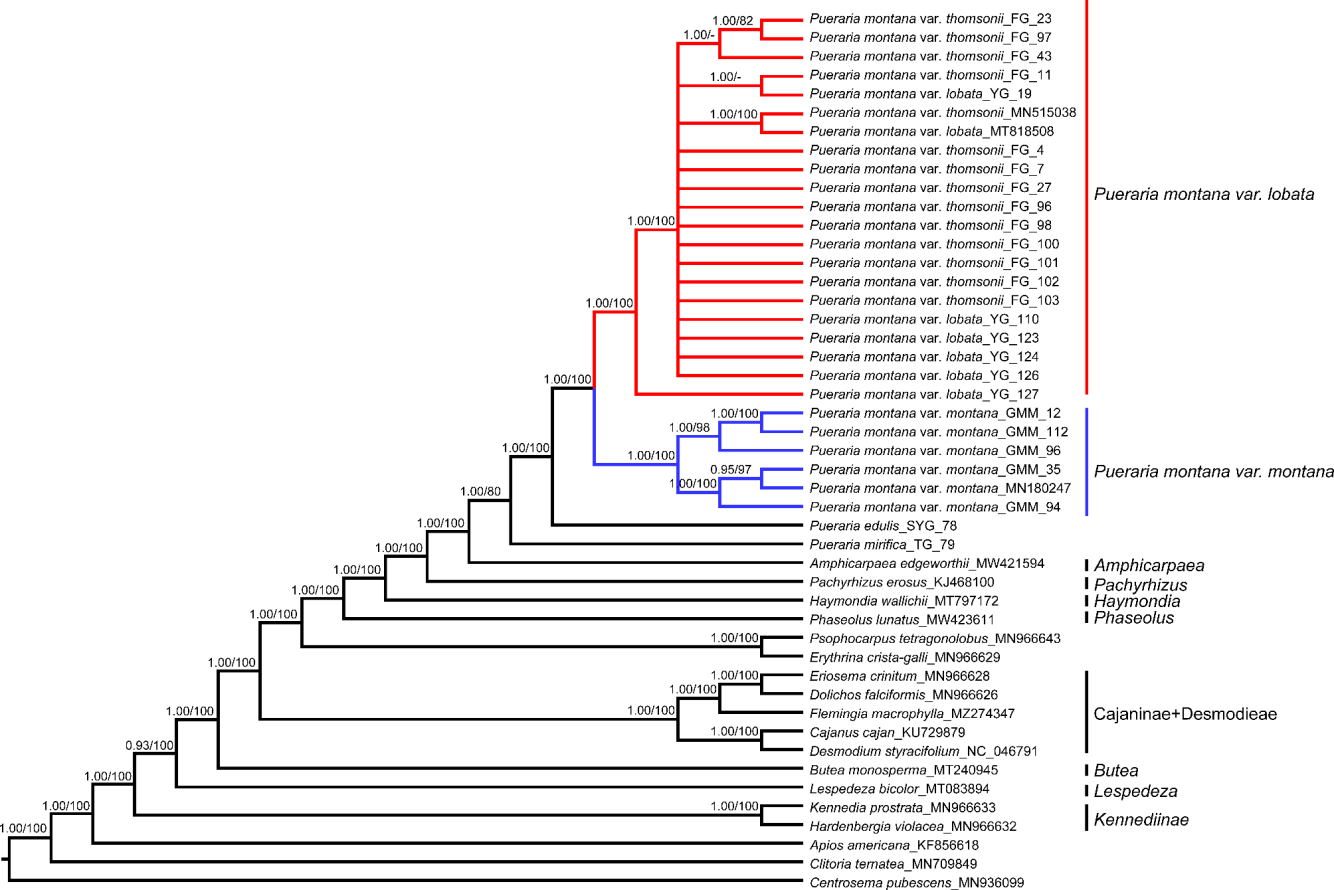
Phylogenetic tree reconstructed based on the 47 complete plastome sequences. Tree topology was based on BI analysis. Numbers at the nodes represent BI posterior probability (PP) / ML bootstrap (MLB) values. PP or MLB values lower than 0.5 or 50% were indicated by hyphens

The multiple sequence alignment of all 47 plastomes (including both newly generated and previously published plastomes) was 154,176 bp after excluding one of the inverted repeat regions. The best-fit model for the chloroplast genomes, SSC, IR, LSC, and coding sequences was listed in Table 2. The aligned sequences of the five sequence datasets were little saturated and were thus useful for phylogenetic analyses (Table S5). The topologies estimated from the 47 complete chloroplast sequences (Fig. 3) were broadly similar to those estimated using the other four datasets (Fig. S6-S9), where two major clades of the accessions of *P. montana* var. *lobata* and closely related taxa were identified. The monophyly of the *Pueraria montana* var. *lobata* clade including all the individuals of *P. montana* var. *lobata* and *P. montana* var. *thomsonii* was strongly supported in all phylogenetic analyses. All sampled individuals of *P. montana* var. *montana* were monophyletic in LSC, SSC and protein-coding sequences dataset and paraphyletic in IR dataset with low support. Thus, we limited the discussion to phylogenetic trees estimated using chloroplast genomes (Fig. 3).

**Table 2:** Data set characteristics and best substitution models

Taxa	Data set	Number of sites	Gaps and missing data (%)	Number of variable sites	Number of informative sites	GC (%)	Best fit model
47	LSC	98,822	18.9	31,993	14,520	32.9	GTR + I + G
47	SSC	22,607	24.1	11,481	7823	28.9	TVM + G
47	IRs	28,585	20.0	11,465	9886	42.5	TVM + G
47	73 protein-coding genes	58,555	25.8	10,978	5463	37.0	GTR + I + G
47	Data-complete	154,176	17.9	49,163	24,596	34.0	GTR + I + G
30	Data-complete in the taxon removal experiments*	129,134	1.7	3269	500	34.1	TVM + I + G

* This dataset only included 30 accessions of *Pueraria montana* var. *lobata* and its closely related taxa.

*Pueraria mirifica* (PP/MLB = 1.00/80) and *P. edulis* (PP/MLB = 1.00/100) was sister to the varieties of *P. montana* with strong support, respectively. *Pueraria montana* var. *lobata* and the other two varieties are divided into two clades. The *Pueraria montana* var. *montana* clade, containing all accessions of *P. montana* var. *montana*, showed high support (PP/MLB = 1.00/100). The *Pueraria montana* var. *lobata* clade, which includes all accessions of *P. montana* var. *lobata* and *P. montana* var *thomsonii*, is a monophyletic lineage with strong support (PP/MLB = 1.00/100), but the resolution among the accessions within this clade was relatively low.

### Topology testing

**Fig. 4:**
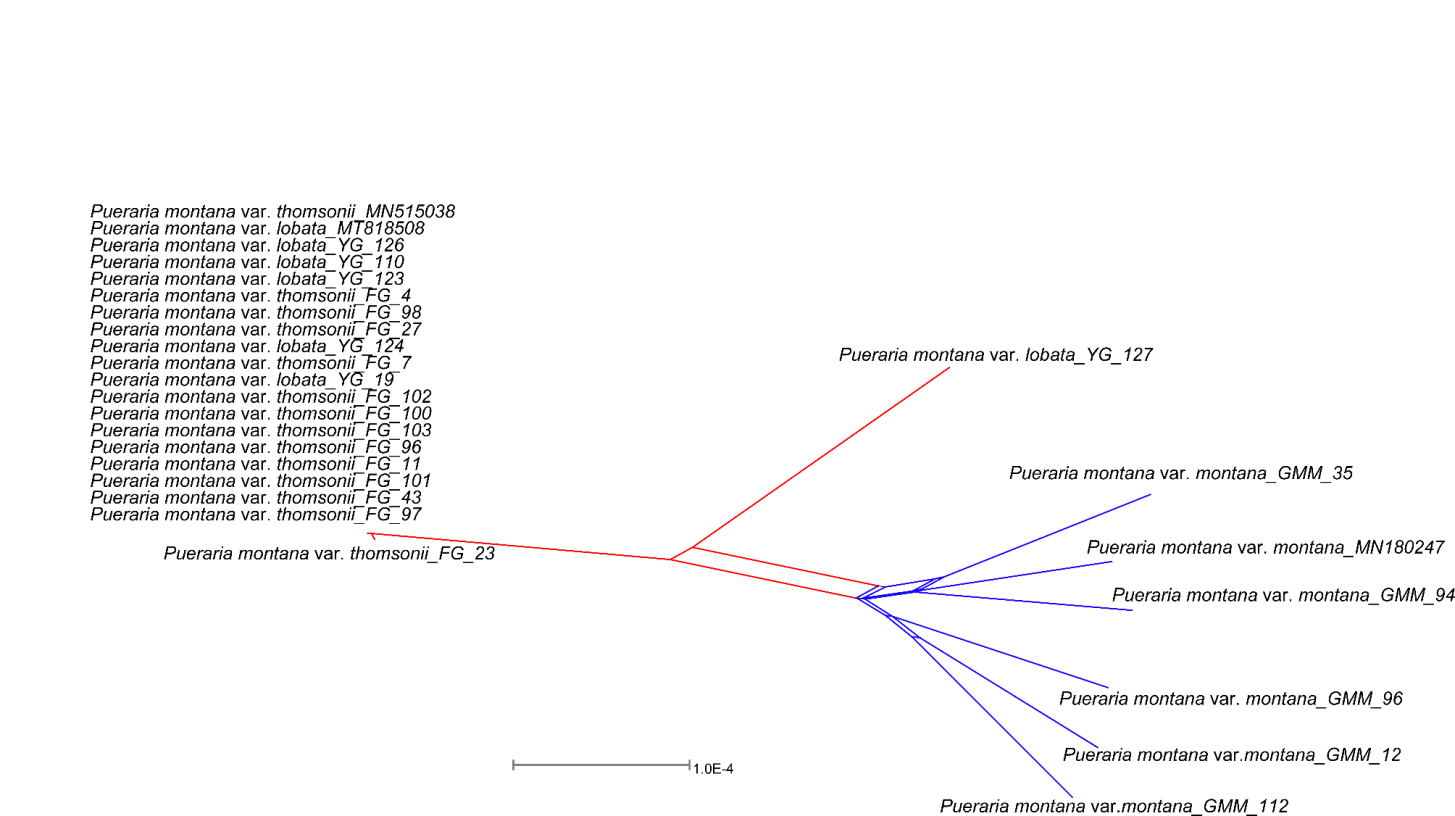
Neighbor-net analyses of *Pueraria* accessions based on 27 plastome sequences. Clades revealed are labeled with different colors

Since the tree topologies of the major clades based on the taxon removal experiments showed no changes when outgroups were removed (Fig. S10), we concluded that the tree topology was not affected by long-branch attraction. The incompletely resolved relationships in the network suggested substantial conflicting signals (Fig. 4). All accessions were sorted into respective clades, and the clades were almost distinct. However, the relationships within the *Pueraria montana* var. *montana* clade were less clear.

### Positive selection

*Pueraria montana* var. *lobata* and the other two varieties from different productive areas were divided into two clades. The branch model detected only one gene (*ycf2*) with a significantly decelerated rate of evolution (ω = 0.62093, *p* < 0.001). We detected one gene (*ndhB*, one site) under positive selection at one amino-acid site (*p* < 0.001, BEB value > 0.95) in the analyses of the branch-site models, taking the *Pueraria montana* var. *lobata* clade as the foreground clade. However, no signals of positive selection were detected in the *Pueraria montana* var. *montana* clade in either model. Because chloroplast genes tend to be conservative, we compared the fit of the Clade model C (CmC) with Likelihood Ratio Tests (LRT) to detect subtle differences in specific selective constraints between the *Pueraria montana* var. *lobata* clade and *Pueraria montana* var. *montana* clade. Our CmC analysis showed that accessions of the *Pueraria montana* var. *lobata* clade exhibited significant relaxation in six genes (*accD*, *ndhB*, *ndhC*, *rpl2*, *rpoC2*, *rps2*), compared to accessions of the *Pueraria montana* var. *montana* clade (Table S6). 26 amino acid residues were identified as having a sign of positive selection in the analyses of the Site model, which predicted that 36 sites greater than 0.95 and 33 sites greater than 0.99 (Table S7) of all legume taxa.

## Discussion

### Phylogenetic inference

Previous studies revealed that *Pueraria s.l*. is a polyphyletic group since several lineages could be treated as five distinct genera, namely *Pueraria* *s.s*., *Teyleria stricta* (Kurz) (A) N. Egan & (B) Pan, *Neustanthus* Bentham, *Haymondia* A.N. Egan & B. Pan, and *Toxicopueraria* A.N. Egan & B. Pan based on several key morphological and phylogenetic studies. The remaining species include *Pueraria tuberosa* (Roxb. ex Willd.) DC. grouped into *Pueraria s.s.* [7, 10–16]. The infrageneric classification of the *Pueraria s.s.* is still largely in dispute, and it is an intricate challenge for taxonomic species, subspecies, or varieties as many synonyms are reported for a single species. For example, *Pueraria edulis* is treated as the kudzu vine in the southwestern region of China such as Sichuan and Yunnan provinces [37]. In previous works, the phylogenetic tree based on two cpDNA markers or one nuclear maker (*AS*2) showed that *P. montana* var. *lobata* and the other two varieties formed a subclade and nested within *Pueraria s.s.* with other closely related *Pueraria* species [16]. Our plastomic data support the previous findings that *Haymondia wallichii* diverges from *Pueraria* taxa and demonstrate *P. edulis* and *P. mirifica* are separated from three varieties of *P. montana*, then further revealing that *P. montana* var. *lobata* and the other two varieties were divided into two clades, of which one was comprised of *P. montana* var. *lobata* and *P. montana* var. *thomsonii*, and the other clade included all sampled *P. montana* var. *montana*.

For the most part, our phylogenetic results revealed that the *Pueraria montana* var. *montana* clade is resolved as a monophyletic group with strong support except for phylogenetic analysis based on IR regions. The phylogenetic analysis based on nrITS sequences showed that *P. montana* var. *montana* formed a monophyletic clade sister to the other two varieties, which suggested that *P. montana* var. *montana* should be treated as a single species [11, 15, 17]. *Pueraria montana* var. *lobata* and *P. montana* var. *thomsonii* were different from *P. montana* var. *montana* in aspects of the morphology of leaflets, the relative length between inflorescence and leaves, the length and hairiness of the pedicel, the relative length between the wing and keel-petal (Table S2) [9, 10]. Furthermore, the composition and abundance of secondary metabolites also showed significant differences in *P. montana* var. *montana* compared to *P. montana* var. *lobata* and *P. montana* var. *thomsonii* [38].

All our phylogenetic analyses support the sister relationship of *P. montana* var. *lobata* and *P. montana* var. *thomsonii* with strong support but the relationship among accessions are less resolved. The uncertain association between *P. montana* var. *lobata* and *P. montana* var. *thomsonii* was also revealed in previous studies [9, 10]. Although these two varieties were having high medicinal values, there are considerable differences in terms of the metabolites, material basis, and efficacy [38–41]. Morphological intermediacy, insufficiently informative characters, artificial domestication, species complex and putative hybridization/introgression contribute to the lack of agreement between morphological-based classification schemes and recent molecular phylogenies among *P. montana* var. *lobata* and closely related taxa [11, 15–17]. Further studies of *Pueraria* species should focus on other markers to achieve better phylogenetic resolution, such as RAD-seq or transcriptome.

### Conservation and adaptation of kudzu plastomes

Our results reveal no obvious expansion or contraction of IR region, indels and inversion in the chloroplast genome between *P. montana* var. *lobata* and closely related taxa, even compared to the outgroup species of *P. mirifica*. The results of the mVISTA analysis showed variable intergenic regions in the LSC region. Lower sequence diversity existed in the SSC and IR regions of 24 newly sequenced three varieties of *P. montana*. The nine noncoding regions (*trnK-rbcL*, *trnG-psbZ*, *psbD-trnT*, *trnD-psbM*, *petN-trnC*, *psbK-trnQ*, *rps16-accD*, *psbB-psbT*, and *rps3-rps19*) and one gene (*ndhJ*) were detected as relatively divergent in the LSC region, while two genes (*rps15*, *ycf1*) and one noncoding region (*ccsA*-*ndhA*) were detected in SSC region. These variable regions identified in the present study could be potential markers for phylogenetic or population genetics studies in *P. montana* var. *lobata* and its related taxa.

*Pueraria* species are delineated as climbing vines. *Pueraria montana* var. *lobata* (kudzu) inhabits temperate environments or higher altitudes in the tropics [7] and prefers to occur in high-light forest edge areas, even though it can live under both shade and sun [34]. Thus, *Pueraria montana* var. *lobata* has been widely found in its native area, and probably introduced elsewhere [7]. The ecology, climatic considerations, growth and establishment pattern, cultivation and genetic divergence may influence the distribution of *P. montana* var. *lobata*, even other *Pueraria* plants [42]. Interestingly, neither similar environment nor genetics seems to be a major factor in the distribution of kudzu, as is the case in the United States and Switzerland [42]. The ecology and genetics of *P. montana* var. *lobata* in Southern Switzerland is similar to that in the South-Eastern USA [42]. *Pueraria montana* var. *lobata* is not expected to spread on a large scale in Southern Switzerland [42, 43], while it was officially listed as a Federal Noxious Weed due to widespread ecological and economic impacts in the southeastern USA [34, 44]. For example, its invasion has the potential to raise ozone pollution in the southeastern United States [45]. To clarify the adaptation or differentiation of *P. montana* var. *lobata* and its closely related taxa, our data provide novel evidence of positive selection of genes under four models from the perspective of the chloroplast genome, as well as in *Panax* [27], and *Rhodiola* [31].

The clade model detected the variation in *accD*, *ndhB*, *ndhC*, *rpl2*, *rpoC2*, *rps2* as being significantly different from the accessions of the *Pueraria montana* var. *montana* clade. The plastid *accD* gene encodes a key enzyme for fatty acid biosynthesis, namely a beta subunit of acetyl-CoA carboxylase, which is important for leaf development [46–48]. The *ndh* genes in plastomes encode a protein act by adjusting the electron transfer from NADH to photosystem I [31, 48]. The gene *rpl2* encodes the ribosomal protein L2 of 80 S ribosomes, which is critical for the peptidyl transferase activity [49]. The gene *rpoC2* encodes RNA polymerase subunits and the gene *rps2* encodes the ribosomal protein, but it is unclear their specific function in the evolution of the plastome of *Pueraria*.

The site model detected 26 amino acid residues across all sampled species. Using the *Pueraria montana* var. *lobata* clade as the foreground, the branch model revealed only *ycf2* may have undergone negative selection. *ycf2*, as the largest coding sequence in plastome and conserved open reading frames, may exhibit similarities with fstH, such as activity associated with cell division, ATPase, and chaperone function [50, 51]. We also detected one possible positively selected gene (*ndhB*) using the branch-site model, which may be involved in photosynthesis and temperature sensitivity [52, 53]. However, taking the *Pueraria montana* var. *montana* clade as the foreground, both the branch and branch-site models failed to detect the positive genes.

The combination of key traits such as relatively high photosynthetic rates, rapid leaf movements, and the ability to fix N_2_ make *P. montana* var. *lobata* an aggressive competitor [34]. Thus, our results propose that the positive selection of these genes, involving leaf development or photosynthesis, may play an important role in adaptation to different habitats in kudzu or other kudzu species, which may influence their plastid differentiation pattern. This adaptation of kudzu and its related taxa occurred when their common ancestor diverged from other *Pueraria* species.

## Conclusions

In this study, we reported the chloroplast genomes for 26 *Pueraria* accessions. Through the comparisons of these newly sequenced chloroplast genomes to additional sequences from species of legume species in NCBI, we found that gene content and order of *P. montana* var. *lobata* and its closely related taxa are highly conserved. The phylogenetic results based on 47 plastomes, LSC, SSC, IR, and protein-coding sequences supported *P. edulis* and *P. mirifica* was sister to remaining *Pueraria* accessions respectively. The remaining *Pueraria* accessions were divided into two clades but could not resolve the further division within these two clades. We also detected several genes that may play the important role in the evolution of these two clades. Overall, these results demonstrated the power of chloroplast genomes in solving the phylogenetic relationship, as well as the variation of chloroplast genomes between *P. montana* var. *lobata* and its closely related species. Future work with more taxa sampling and markers will help to illustrate gene introgression, ILS, and other biological factors that may play various roles among lineages of *P. montana* var. *lobata* and *P. montana* var. *montana*.

## Methods

### Taxon sampling, DNA extraction and sequencing

Three to five fresh leaf samples from each individual were collected in their native habitats and dried in silica gel and stored at room temperature for DNA extraction. Then we selected these accessions from representative productive areas. Twenty-six accessions, including *P. montana* var. *lobata* and the other two varieties, were selected for Illumina sequencing. *Pueraria mirifica* Airy Shaw & Suvat. and *Pueraria edulis* Pampan. were also sequenced as outgroups. All vouchers were deposited in the Guangxi Academy of Agricultural Sciences (GAAS, Nanning, China) (Table 1). The samples of *Pueraria* accessions were identified by Hua-Bing Yan and Xiao-Hong Shang in the field. Silica-dried leaf material was sent to the Novogene Bioinformatics Technology Co. Ltd. (Tianjin, China) for library preparation and Illumina sequencing on NovaSeq 6000. A paired-end library (150 × 2) was constructed with an insert size of 350 base pairs (bp). Total genomic DNA was isolated using a DNeasy Plant Mini Kit (Qiagen, CA, United State).

### Sequence assembly and annotation

The raw data were first filtered using fastp to remove those paired reads that contained more than 10% of N bases, and more than 50% of bases with low Phred quality (Q ≤ 5), and adapter sequences. The clean data were mapping assembly to create initial reference sequences using MIRA v4.0.2 [54]. These newly created reference sequences were assembled as the complete plastome sequences using MITObim v1.8 [55]. The previously published complete plastome sequences of *P. montana* var. *lobata* (GenBank nos., MT818508 and MN180247) and *P. montana* var. *thomsonii* (GenBank no., MN515038) were used as seed references. These assemblies were manually remapped and inspected using Geneious v. R9.0.2 [56] under default parameters to check for misassemblies. The tRNAscan-SE ver. 1.21 software [57] was applied to verify the tRNA genes with default parameters. Gene annotations were assigned using DOGMA [58]. Inverted repeat boundaries were determined using the BLAST method [59] (https://blast.ncbi.nlm.nih.gov/Blast.cgi) and verified using Geneious v. R9.0.2. The circular maps of newly sequenced chloroplast plastomes were drawn using the online program OGDRAW v. 1.2 [60].

### Identification of the hypervariable regions

To better identify possible rearrangements, five newly sequenced chloroplast genomes of *P. montana* var. *lobata* and closely related taxa were compared using the global alignment algorithm Shuffle-LAGAN [61] with mVISTA (https://genome.lbl.gov/vista/mvista/submit.shtml), with plastome of *P. mirifica* was chosen as the reference genomes to detect the gene content and structural variation between *P. montana* var. *lobata* and the other two varieties. To further identify specific patterns of structural variation at the species level, five chloroplast genomes were used as the representatives to align with default parameters using MAUVE plugin [62] in Geneious v R9.0.2. We also detected the contraction and expansion of the boundaries between SC and IR regions using IRscope [63]. The nucleotide diversity (π) analysis of 24 newly sequenced plastomes of three varieties of *P. montana* was implemented using DnaSP v. 6.12.03 [64] with window length set to 500 bp, and the step size set as 200 bp.

### Phylogenetic analyses

To better determine the phylogenetic position of *P. montana* var. *lobata* and other varieties, we reconstructed a phylogeny using 47 chloroplast genomes, including the representatives of the major subclades of legume species referring to the previous studies [16, 18–20]. Twenty-one previously published plastomes were downloaded from the NCBI GenBank database (https://www.ncbi.nlm.nih.gov/) (Table S8). The phylogenetic analyses were conducted based on the following five datasets: (1) the complete chloroplast genome sequences that excluded one copy of the inverted repeat (IR) region; (2) the large single-copy region (LSC); (3) the small single-copy region (SSC); (4) one inverted repeats region (IR), and (5) 73 protein-coding genes excluding genes *infA*, *rpl33*, *rps16*, *ycf1*, *ycf4* that were lost in several outgroups (Table S8). These regions were aligned in Geneious v R9.0.2 using Mauve progressive using default options. DAMBE v6.4.29 was used to assess substitution saturation [65, 66]. Phylogenetic analyses were inferred using both maximum likelihood (ML) and Bayesian inference (BI) via the CIPRES Science Gateway server (http://www.phylo.org/) [67]. ML analysis using rapid bootstrapping and 1000 replications was performed in RAxML [68] with the GTR-GAMMA model. The appropriate model for each dataset was determined using jModelTest v2.1.4 [69]. For the BI analysis, two independent Markov chain Monte Carlo (MCMC) runs with three heated and one cold chain were executed using MrBayes v3.2.7 [70]. Trees were sampled every 1000 generations. The first 25% of trees were discarded as burn-in and the remaining samples were then summarized and a majority-rule consensus tree was constructed. Two million generations were run for the IR dataset and data-complete in the taxon removal experiments dataset, whereas three million generations were run for the other four datasets (Table 2). The stationarity was considered to be reached when the average standard deviation of split frequencies fell below 0.01. We also determined the convergence of runs using Tracer v 1.6 [71] to assess the effective sample size (ESS) > 200 for all parameters.

### Topology hypothesis testing

Taxon removal experiments can aid in the investigation of whether distantly related outgroups have a biased attraction to long branches within the ingroups [72–74]. To test if outgroup selection affected the topology of the ingroups, we recovered phylogenetic trees by removing outgroups to include only 30 accessions of *P. montana* var. *lobata* and its closely related taxa. *Haymondia wallichii* (DC.) (A) N. Egan & (B) Pan bis was chosen as the outgroup based on similar morphology and relatedly phylogenetic relationship with *Pueraria* taxa in our analysis. To better visualize and evaluate the phylogenetic signal conflicts between ingroup taxa, the neighbor-net algorithm based on uncorrected *P*-distances was implemented in SplitsTree v4.14.4 [75], excluding all outgroup taxa to leave *P. montana* var. *lobata* and the other two varieties.

### Positive selection analysis

We chose the ML tree based on 47 chloroplast genomes to identify positively selected genes in the EasyCodeML [76] with the likelihood ratio test (LRT) since the topology of ML trees and BI trees were congruent. Because the genes *infA*, *rpl33*, *rps16*, *ycf1* and *ycf4* were lost in several legume species (Table S8), we selected 73 protein-coding genes for further analysis. The amino acid sequences of the 73 protein-coding genes were aligned using MAFFT and converted nucleotide alignments into PAML format in EasyCodeML according to the manual [76]. Four different models (i.e. site model, branch model, branch-site model, and clade model) were used to test the selection pressure. We set two clades to detect positive evolution at the clade level: one is the *Pueraria montana* var. *lobata* clade and the other is the *Pueraria montana* var. *montana* clade using the other three model selections, namely branch model, branch-site model and clade model. Firstly, we used a branch model to identify rapidly evolving genes. Genes with *p* < 0.05 and ω > 1 are considered under positive selection, while others with *p* < 0.05 and 0 < ω < 1 are considered under purifying selection. The branch-site models were combined with heterogeneous ω across sites and branches to find instances of neutral evolution and negative selection. The clade model was performed to identify subtler differences in site-specific selective constraint among partitions of a phylogeny [77].

## Electronic supplementary material

Below is the link to the electronic supplementary material.

Supplementary Material 1

Supplementary Material 2

## Data Availability

The assembled sequences described in this study have been deposited in the National Center for Biotechnology and Information (NCBI) under the accessions as summarized in Table 1. The phylogenetic genome datasets used and analyzed in this study were retrieved from the National Center for Biotechnology Information Organelle Genome Resource Database.

## Abbreviations

BI: Bayesian interference
IR: Inverted repeat
LRT: Likelihood ratio test
LSC: Long single copy
LSR: Long sequence repeat
MCMC: Markov chain Monte Carlo
ML: Maximum likelihood
PP: Posterior probability
NCBI: National Center for Biotechnology Information
π: nucleotide diversity
rRNA: ribosomal RNA
SSC: Short single copy
tRNA: transfer RNA
