# Supplementary material for: Comparative plastomes of Pueraria montana var. lobata (Leguminosae: Phaseoleae) and closely related taxa: insights into phylogenomic implications and evolutionary divergence
Source: BMC Genomics. 2023 Jun 2;24:299. doi: 10.1186/s12864-023-09356-8 (PMC10239116; doi:10.1186/s12864-023-09356-8)
Supplement: Supplementary file 2 — Supplementary Material 2 [file 12864_2023_9356_MOESM2_ESM.pdf]

Supplementary Figure 1

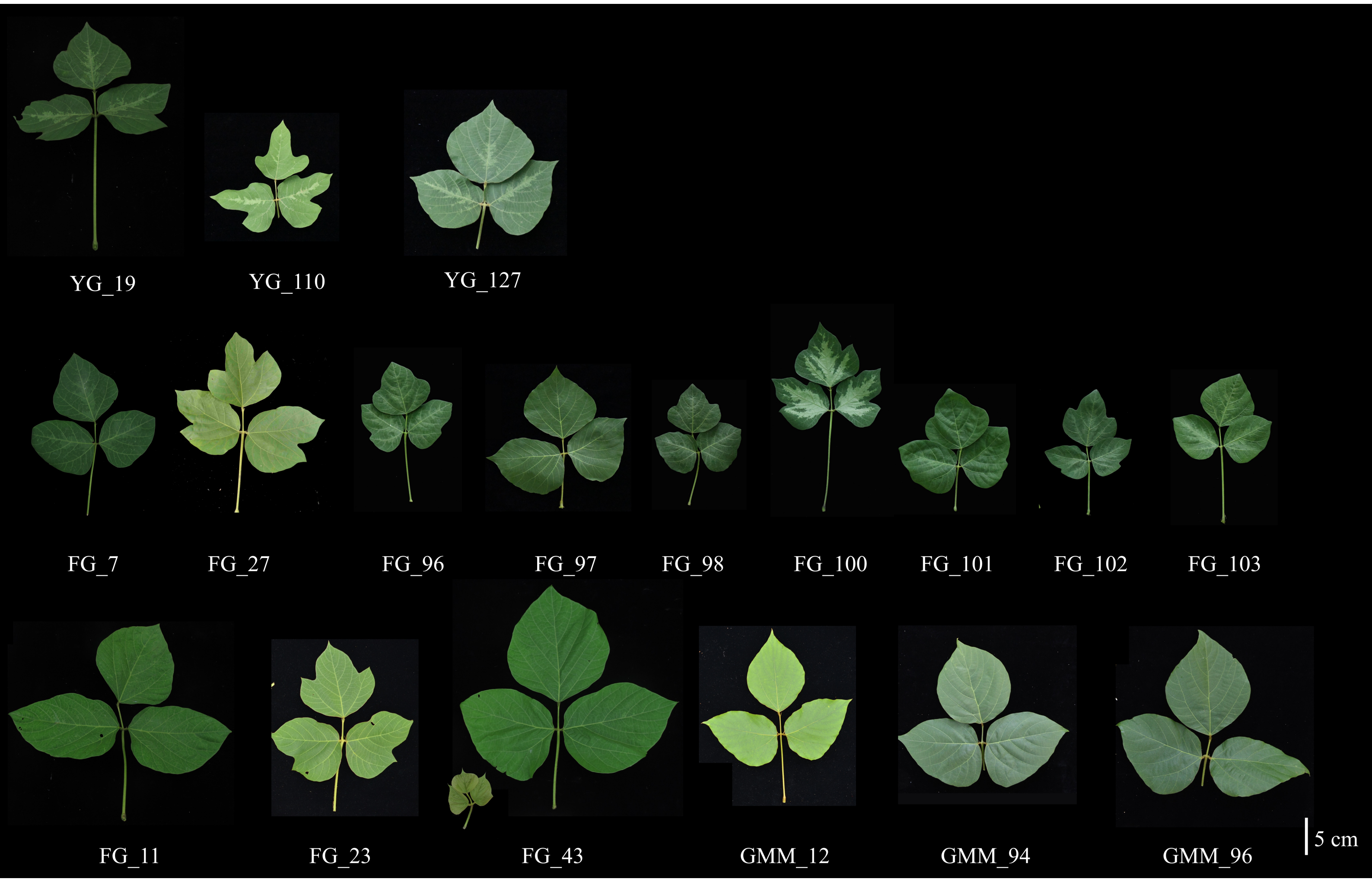

Figure S1 The morphology of the terminal leaf of *Pueraria* accessions in this study. YG: *P. montana* var. *lobata*; FG: *P. montana* var. *thomsonii*; GMM: *P. montana* var. *montana*. The code is consistent with Table 1.

Supplementary Figure 2

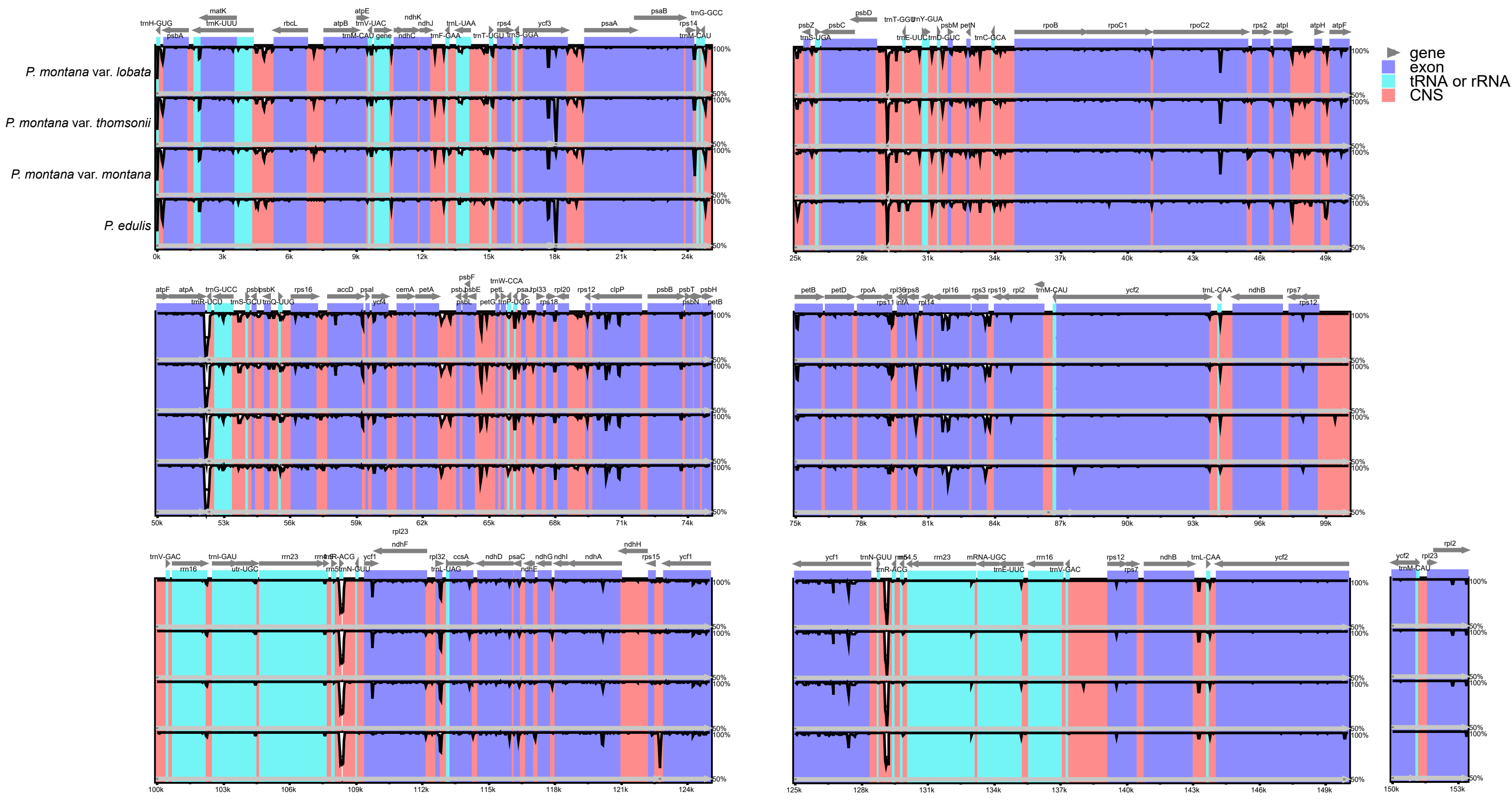

Supplementary Figure 2. Whole-genome alignment of five newly sequenced plastome sequences of *Pueraria* accessions in this study. Sequences of chloroplast genomes were aligned and compared using the mVISTA program. The vertical scale indicates the percentage identity, ranging from 50% to 100%. The chloroplast genome of *P. mirifica* (TG_79) is the reference genome.

Supplementary Figure 3

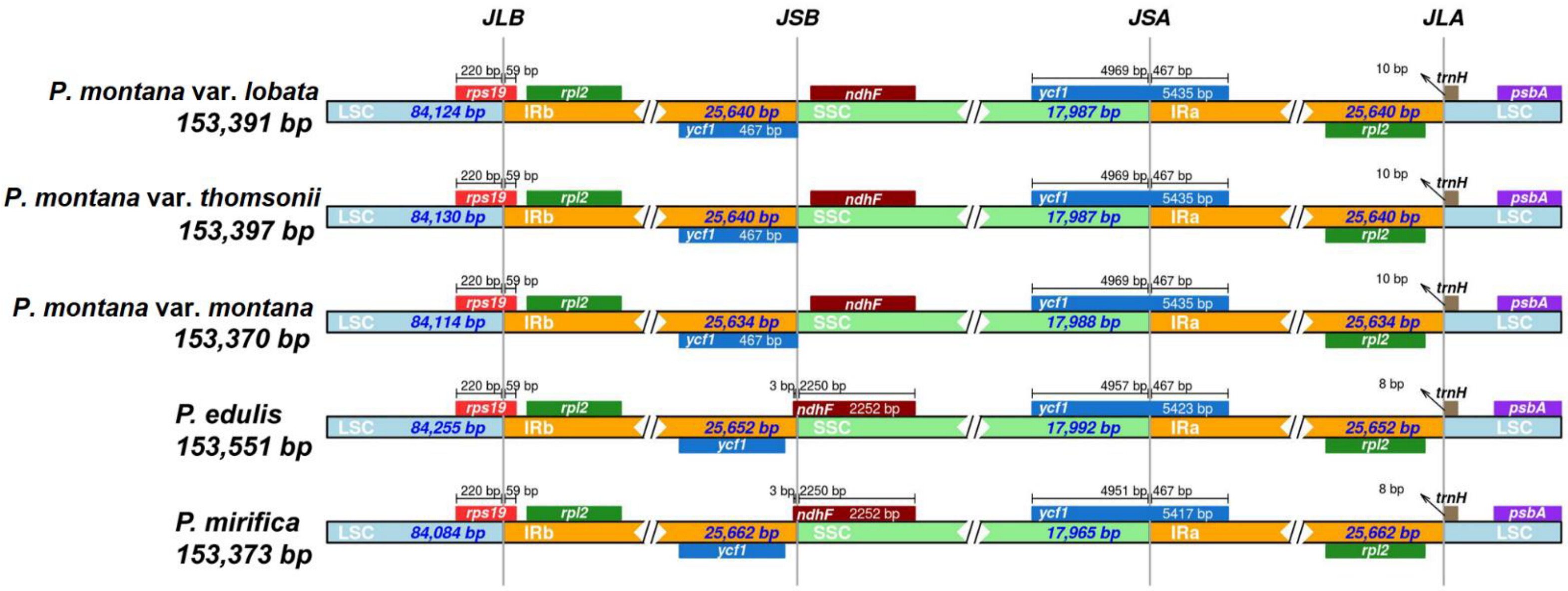

Supplementary Figure 3. Comparison of the borders of IR and SC regions among five newly sequenced plastome sequences of *Pueraria* accessions in this study. Boxes above and below the primary line indicate the adjacent border genes.

Supplementary Figure 4

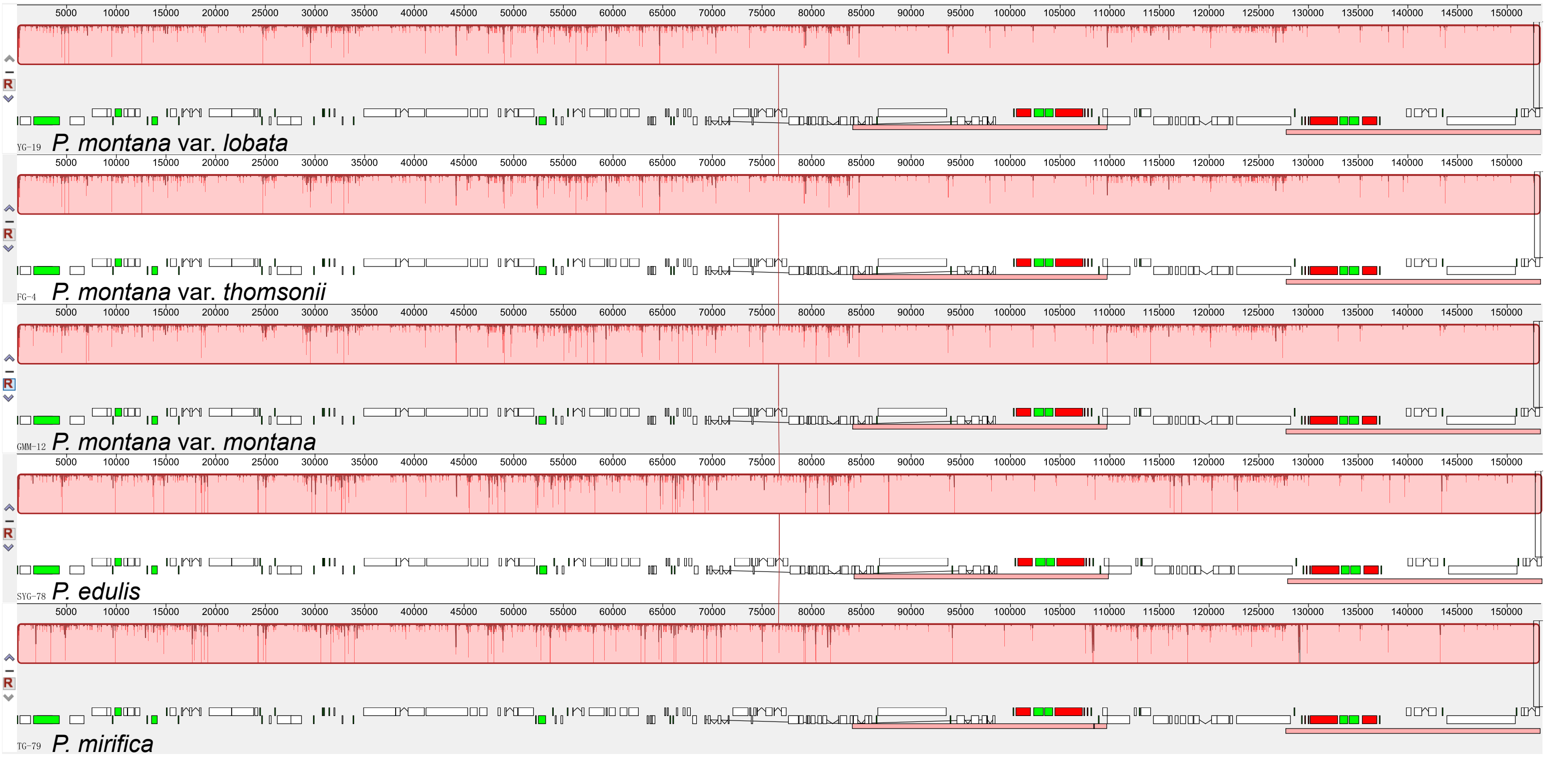

Supplementary Figure 4. The alignment of five newly sequenced plastome sequences of *Pueraria* accessions in this study. The height of the colored region with a block reflects the average sequence identity relative to the reference genomes of *P. mirifica* (TG_79). The small blocks of various colors represent genes. Black represents transfer RNA (tRNA); red represents ribosomal RNA; white represents proteincoding; green represents intron-containing tRNA.

Supplementary Figure 5

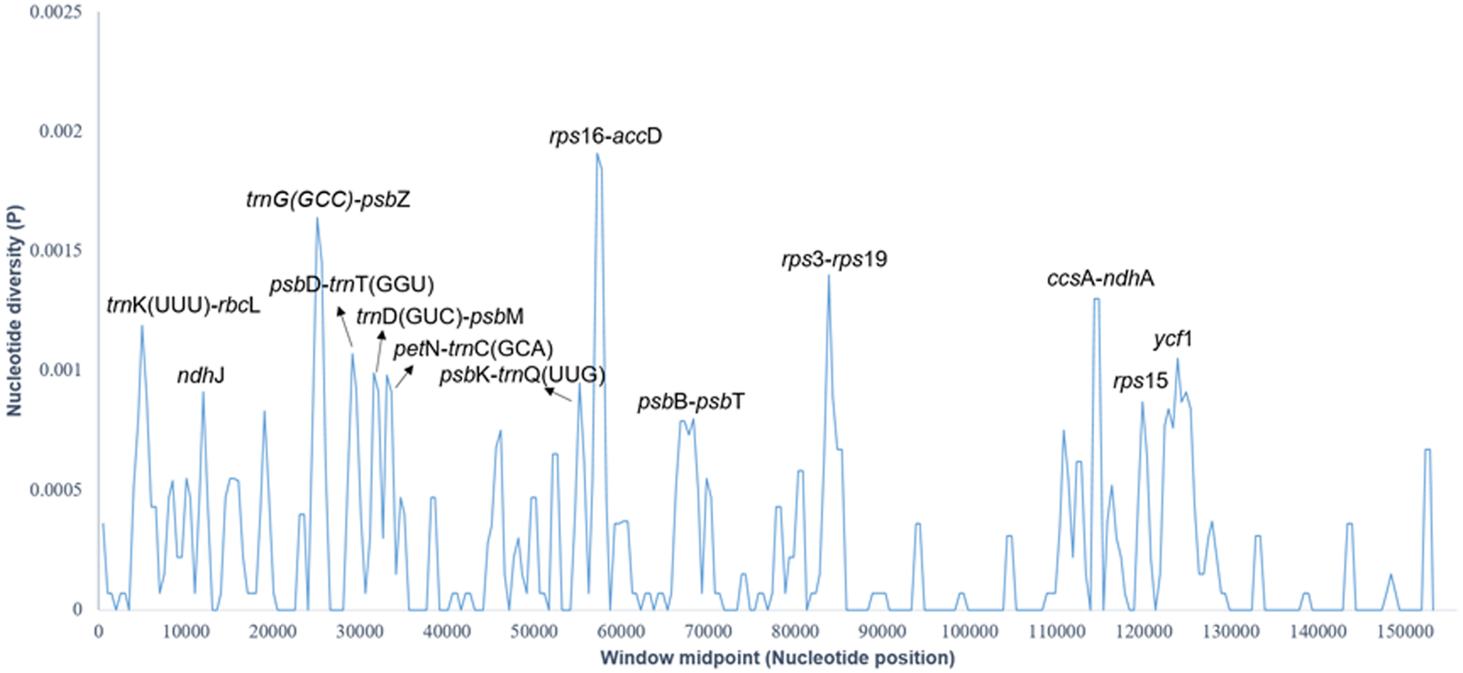

Supplementary Figure 5. Nucleotide diversity (π) in 24 newly sequenced plastomes of three varieties of *P. montana*. Sliding window analysis with a window length of 1000 bp and a step size of 500 bp.

Supplementary Figure 6

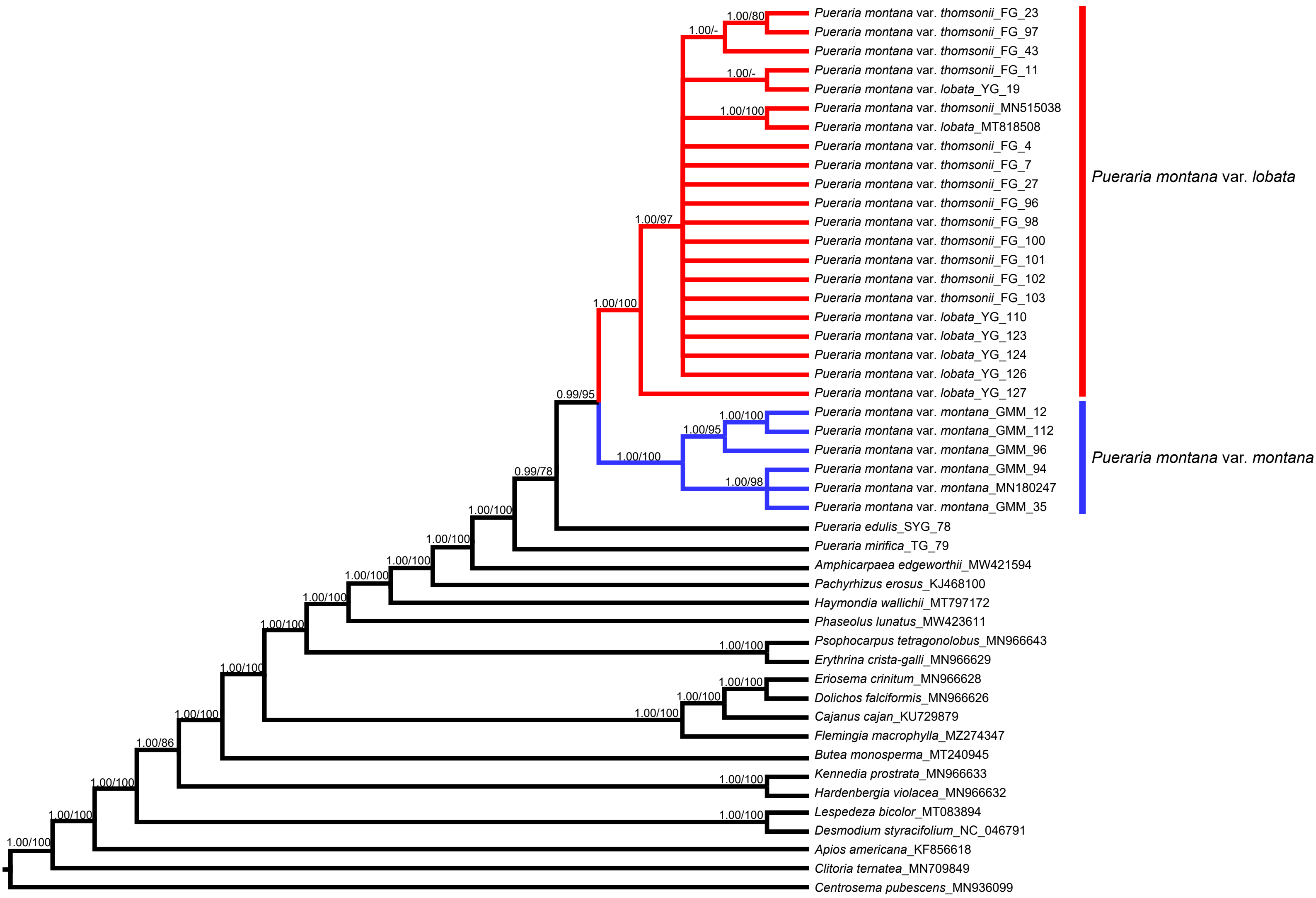

Supplementary Figure 6. Phylogenetic tree reconstructed based on the LSC using Bayesian interference (BI) and maximum likelihood (ML) methods. Numbers at the nodes represent BI posterior probability (PP) / ML bootstrap (MLB) values. PP or MLB values lower than 0.5 or 50% were indicated by hyphens.

Supplementary Figure 7

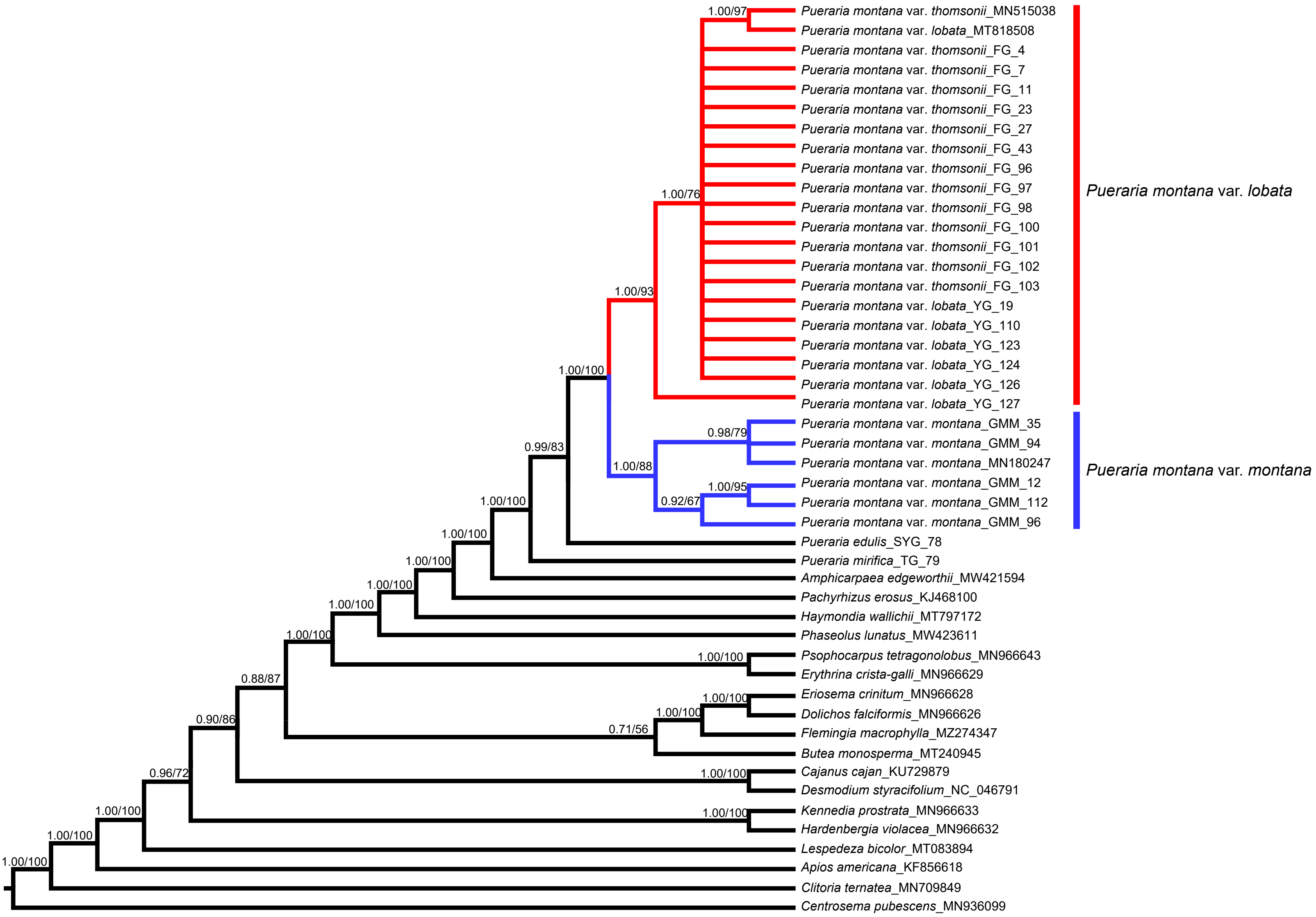

Supplementary Figure 7. Phylogenetic tree reconstructed based on the SSC using Bayesian interference (BI) and maximum likelihood (ML) methods. Numbers at the nodes represent BI posterior probability (PP) / ML bootstrap (MLB) values. PP or MLB values lower than 0.5 or 50% were indicated by hyphens.

Supplementary Figure 8

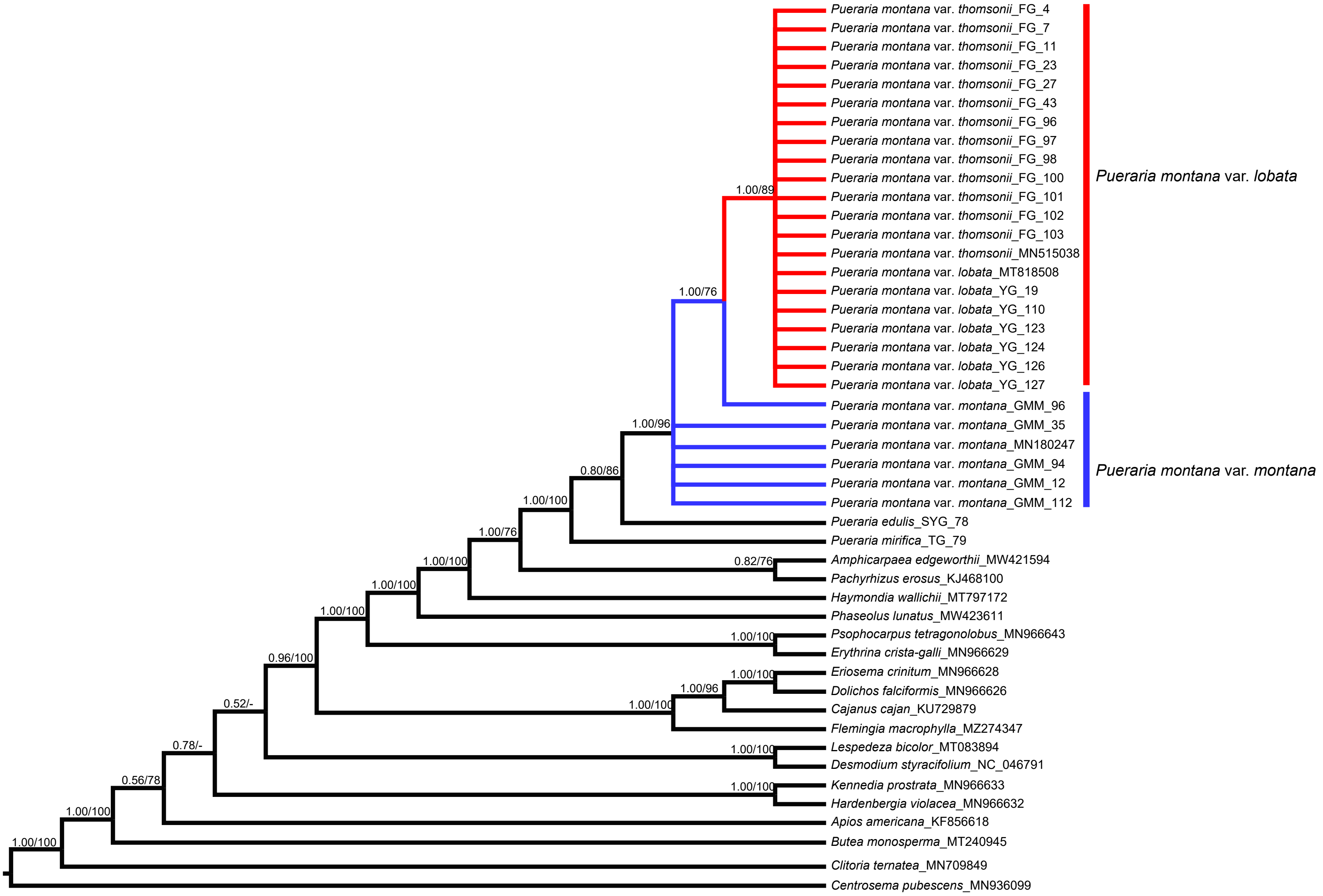

Supplementary Figure 8. Phylogenetic tree reconstructed based on the IR using Bayesian interference (BI) and maximum likelihood (ML) methods. Numbers at the nodes represent BI posterior probability (PP) / ML bootstrap (MLB) values. PP or MLB values lower than 0.5 or 50% were indicated by hyphens.

Supplementary Figure 9

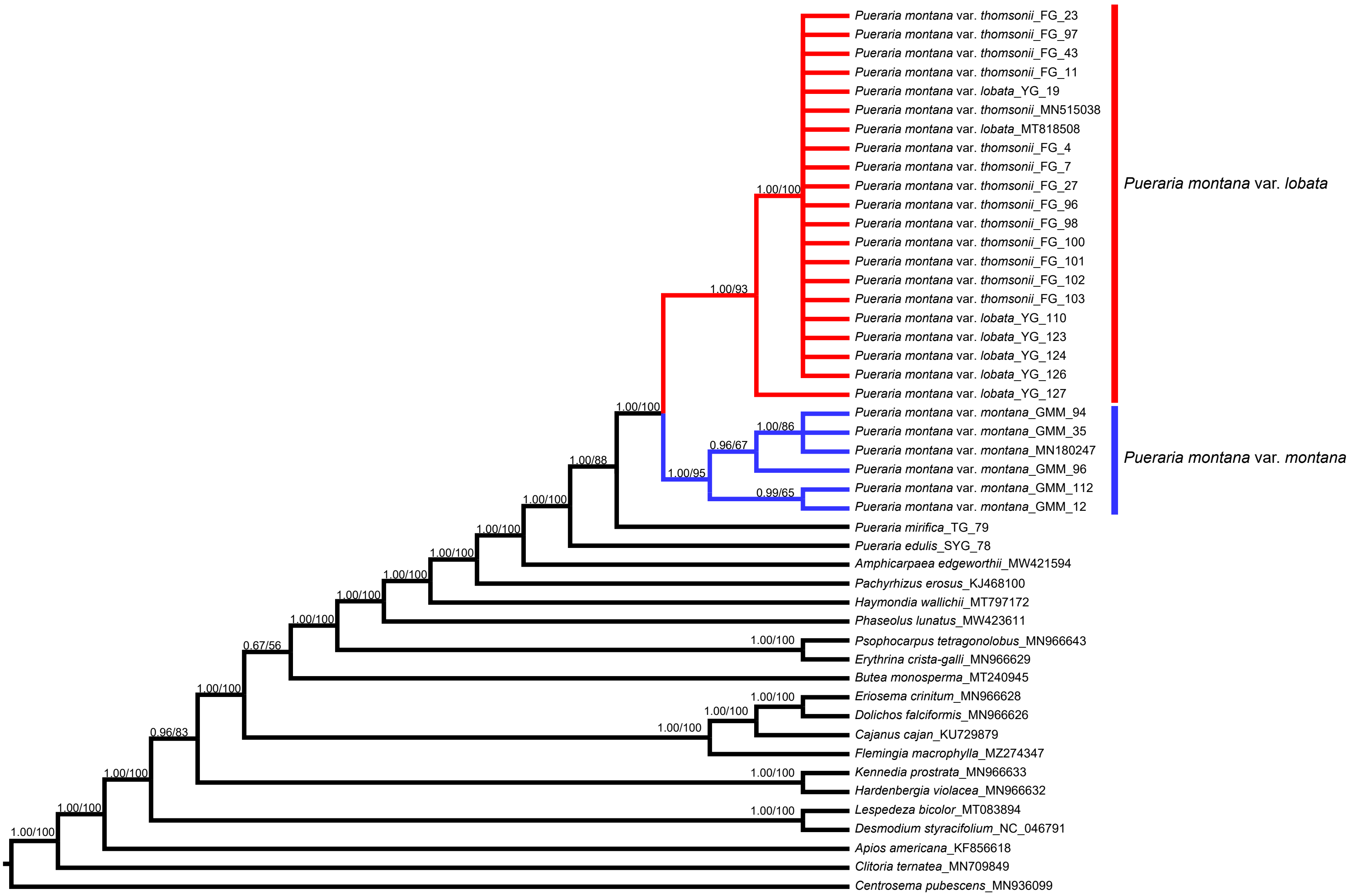

Supplementary Figure 9. Phylogenetic tree reconstructed based on the 73 protein-coding genes using Bayesian interference (BI) and maximum likelihood (ML) methods. Numbers at the nodes represent BI posterior probability (PP) / ML bootstrap (MLB) values. PP or MLB values lower than 0.5 or 50% were indicated by hyphens.

Supplementary Figure 10

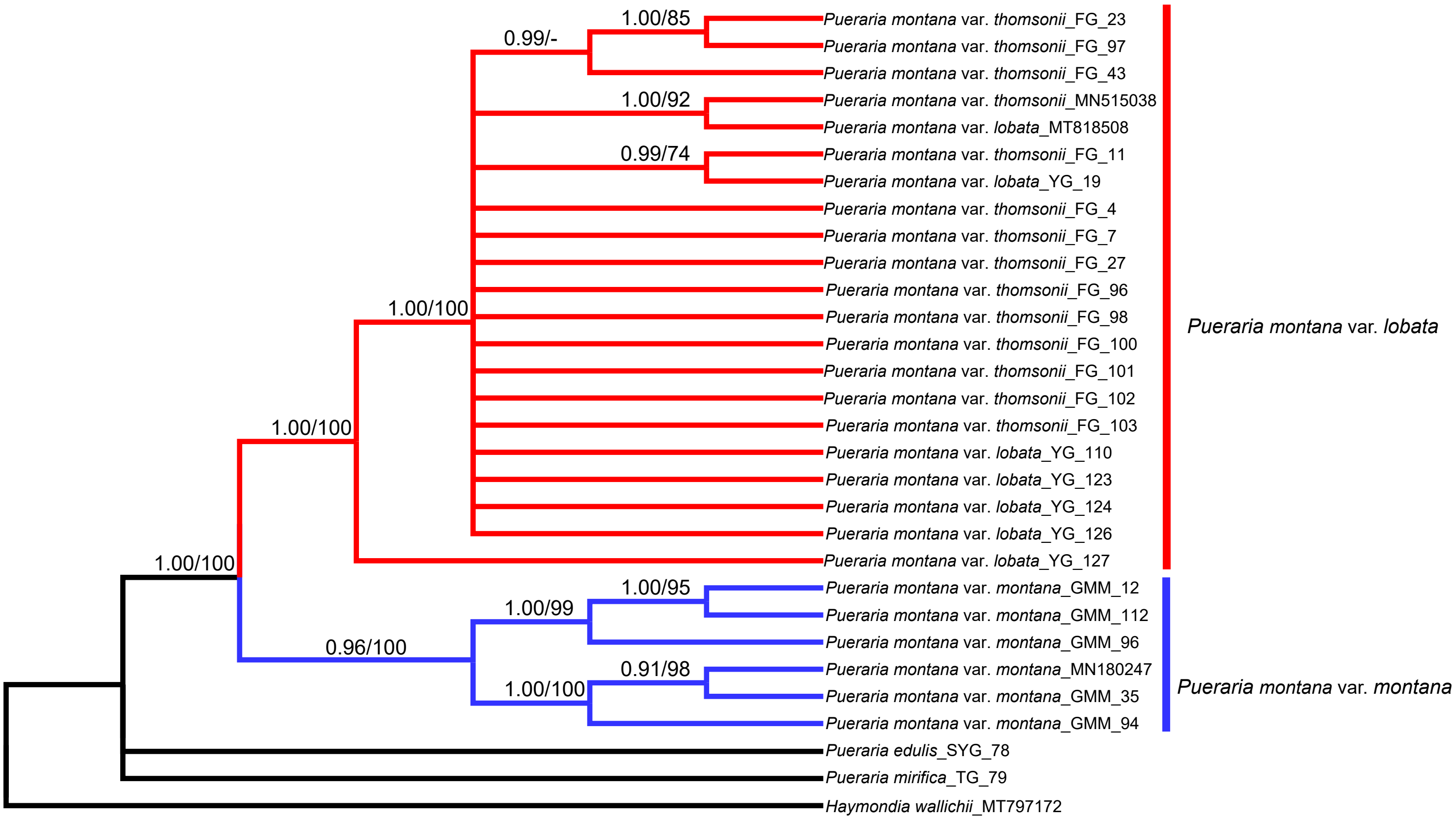

Supplementary Figure 10. Phylogenetic tree reconstructed based on the 30 complete plastomes sequences using Bayesian interference (BI) and maximum likelihood (ML) methods. Numbers at the nodes represent BI posterior probability (PP) / ML bootstrap (MLB) values. PP or MLB values lower than 0.5 or 50% were indicated by hyphens.
